# Improving HIV medical care engagement by attending to status disclosure and social support

**DOI:** 10.1080/09540121.2020.1718588

**Published:** 2020-01-28

**Authors:** Neal A. Carnes, James W. Carey, Deborah J. Gelaude, Damian J. Denson, Patricia A. Bessler

**Affiliations:** Division of HIV/AIDS Prevention (DHAP), National Center for HIV/AIDS, Viral Hepatitis, STD, and TB Prevention (NCHHSTP), Centers for Disease Control and Prevention (CDC), Atlanta, GA, USA

**Keywords:** Disclosure, social support, HIV medical care engagement, black men who have sex with men, Latino men who have sex with men

## Abstract

Expeditious linkage and consistent engagement in medical care is important for people with HIV’s (PWH) health. One theory on fostering linkage and engagement involves HIV status disclosure to mobilize social support. To assess disclosure and social support’s association with linkage and engagement, we conducted a qualitative study sampling black and Latino men who have sex with men (MSM of color) in the U.S. Participants’ narratives presented mixed results. For instance, several participants who reported delaying, inconsistent access, or detachment from care also reported disclosing for support purposes, yet sporadic engagement suggests that their disclosure or any subsequent social support have not assisted. The findings contribute to the literature that questions disclosure and social support’s influence on care engagement, especially when decontextualized from circumstances and intentions. Our findings suggest the mechanics of disclosure and social support require planned implementation if intending to affect outcomes, especially among MSM of color. From the findings, we explore steps that may bolster interventions seeking to anchor medical care engagement.

## Introduction

Early and consistent medical care is essential for optimizing people with HIV’s (PWH) health (Crawford & Thornton, 2017; Hall et al., 2016; Liau et al., 2013; Robertson, Laraque, Mavronicolas, Braunstein, & Torian, 2015). Some PWH, however, delay or avoid medical care (CDC, 2017). Compared to white men who have sex with men (MSM), black MSM experience a disparity in linkage to and retention in care and a disproportionate number of Latino MSM delay entry into care (Dasgupta, 2016; Laffoon et al., 2015; Singh, Mitsch, & Wu, 2017). Christopoulos and colleagues (2011) found that effectively linking and retaining black and Latino MSM often involves community and personal network resources.

One theory argues that support network resources, e.g., friends and family, can assist PWH with care engagement (Cook, Canidate, Ennis, & Cook, 2018; Geter, Sutton, & Hubbard McCree, 2018). The theory asserts when PWH disclose their status they build a support network. This network provides emotional, informational, or instrumental means, which mediate care engagement (Smith, Rossetto, & Peterson, 2008; Valle & Levy, 2009; Waddell & Messeri, 2006). In accordance with this theory, interventions such as HIV Navigation Services, promote status disclosure for social support purposes (Chaudoir, Fisher, & Simoni, 2011; Dima, Stutterheim, Lyimo, & de Bruin, 2014; Elopre et al., 2015; Obermeyer, Baijal, & Pegurri, 2011; Overstreet, Earnshaw, Kalichman, & Quinn, 2013; WHO, 2013).

Some research primarily affirms the theory (Elopre et al., 2015; Strachan, Bennett, Russo, & Roy-Byrne, 2007; WHO, 2013). For example, in a study of black and Latino PWH, Wohl and colleagues (2011) found an association between retention in care and HIV status disclosure, when the PWH disclosed to a greater number of people. While other studies primarily refute the theory (Daskalopoulou et al., 2017; Hamilton, Razzano, & Martin, 2007; Mellins, Kang, Leu, Havens, & Chesney, 2003; Nakigozi et al., 2015; Thompson et al., 2012). Kelly and colleagues (2014) found that social support did not predict retention in care yet did correlate with linkage.

The purpose of this paper is to: (1) assess disclosure and social support experiences in context to black and Latino MSM’s degree of HIV medical care engagement; and, (2) discern how to enhance intended outcomes when encouraging disclosure for social support purposes.

## Materials and methods

We conducted a qualitative study sampling from Atlanta, Baltimore/Washington DC, Chicago, and Los Angeles. Recruitment occurred through passive measures, e.g., flyers. Participants self-reported eligibility criteria, which included living with HIV, 18 years of age or older, male, black and/or Latino, sex with another man in the prior six months, and spoke English or Spanish. Emory University’s Institutional Review Board approved the study protocol.

A single session, semi-structured, individual interview was conducted with 84 men living with HIV. On average, the interviews lasted an hour and were conducted by trained qualitative researchers in a private setting. Researchers audio-recorded and transcribed the recordings verbatim. We translated Spanish language transcripts for analytic purposes. To ensure consistent application of the coding scheme, coding was finalized after attaining acceptable intercoder reliability (i.e., kappa scores ≥ .70). We used NVivo^®^ to code, and SPSS 21^®^ to generate frequencies.

Interview questions were grouped thematically: (1) demographics; (2) understanding lab tests; (3) experiences with treatment planning; (4) experiences with providers and healthcare facilities; (5) barriers and facilitators to care and treatment; and, (6) status disclosure and social support. We focused these analyses on the participants’ degree of care engagement since diagnosis in association with HIV disclosure and social support.

### Engagement status

We found participants’ engagement clustered around four statuses: *engaged*, *delayed*, *inconsistent*, and *detached*. *Engaged* meant the participant reported seeing a provider consistently since diagnosis, whereas *delayed* meant they had a period after diagnosis where they did not see a provider, yet they have subsequently entered care and remained engaged since. *Inconsistent* meant they reported periods in and out of care, while *detached* meant they have not seen a provider with any consistency. Our rationale noted that if disclosure and social support influence engagement, variation between statuses should reflect different experiences with disclosure and social support.

To discern patterns, we grouped participants’ disclosure and social support narratives according to their engagement status. To help ensure a more robust understanding of disclosure and social support, we also inspected response frequencies for select thematic codes: (1) the number of person roles, e.g., “friends”, the participant reported disclosing their status; (2) time between diagnosis and first disclosure; and, (3) disclosure for support in “managing their care”. The codes were defined prior to the development of the status categories; thus, they did not bias the codes. Only the person role code resulted from a structured question; the other themes emerged as *in vivo* responses to probes, thus not all participants registered a response for these codes.

## Results

### Participant characteristics

Participant (*n* = 84) distribution by city was: 29% Atlanta, 27% Chicago, 24% Los Angeles, and 20% Baltimore/Washington DC (Table 1). Slightly more than half (60%) identified as black, 37% as Latino, and 4% as black-Latino. The mean age was 42.4 years old (SD = 10.3). Approximately one-third (32%) were HIV “long term survivors”, meaning they were diagnosed prior to 1997. Three-quarters (76%) reported either full (e.g., Medicaid) or partial (e.g., Ryan White) healthcare coverage. Regarding engagement status, 44% were classified *engaged*, 18% *delayed*, 33% *inconsistent*, and 5% *detached*.

The following summarizes the disclosure and social support narratives by engagement status.

### Engaged

Supporting the theory, one care engaged participant stated, “All my family and friends know”. Another stated, “I am very open and up front with my status”. On the other hand, several engaged participants limited disclosure; “I’m comfortable talking to some of my friends about my HIV status; not my family”. “I haven’t talked to anybody personal, like family”. “I don’t talk about it because it’s not like they can do anything about it … when my close friends, who are physicians, then, you know, of course, that’s a different conversation”. Some were even more restrictive; “I rather keep my status private”. Across the engaged participant narratives, some told many people, and some were selective. Regardless of volume, these men reported being engaged in care since diagnosis. This suggests that if disclosure volume benefits engagement, it is not uniform.

Of the 37 men reporting engaged care, 12 noted “assistance with managing their care” as motivating disclosure. Of the 12, four reported disclosing for assistance in emergencies. Disclosing for emergencies suggests that even when health reasons motivate disclosure, it is not necessarily for the chronic aspects of HIV care. We highlight this difference to affirm the complex nature of disclosure and social support – some sought long-term assistance, while others were more episodic in perspective. Additionally, some disclosed based on the nature of the relationship. “I better tell the manager at work. If something happens, he shouldn’t think I’m playing”. Whatever the motivation, these excerpts tell us that disclosure is complex and can be relationship-dependent as much as need based.

A plurality of engaged participants reported they disclosed to a select group of people. Further, only five reported that they disclosed in the first year after diagnosis, and three delayed disclosing for over a year, thus their linkage to care did not appear predicated on disclosure or social support. In sum, we observed no discernable pattern that supports an association between disclosure and social support and that these men linked to care shortly after diagnosis and have remained engaged since.

### Delayed

Delayed participants also offered a range of disclosure and support experiences. One participant stated, “When I became infected, I had to tell them [family and friends]”. He disclosed shortly after diagnosis. Based on his delay into care, this early disclosure and a potential social support system did not result in expeditious linkage. Another delayed participant also discussed disclosing shortly after diagnosis based on the ethos, “If you [are] honest … you get more respect … the truth will always set you free”. This participant’s early disclosure ethos, also, did not result in expeditious linkage.

The following delayed participant discussed an evolving approach to disclosure, “I really don’t tell people *now* … it’s none of their business”, (emphasis added). This participant reported disclosing shortly after diagnosis yet has refrained since. His timeline does not support the theory in that early disclosure did not result in linkage. However, there were narratives that supported the theory. When one delayed participant disclosed to his friends, their support and encouragement compelled him to seek care. He stated, “I told my best friend and another good friend … they are actually the reason why I went to the emergency room to try to get treatment”.

Like some of the engaged participants, several delayed men disclosed for emergency purposes. One man stated, “I told one sister, and she said, ‘why are you telling me this?’ I said, ‘well, if I get sick, somebody needs to know’”. This excerpt suggests motivations to disclose were similar across groups, yet with different outcomes. In support, another delayed participant stated, “I like putting it out there … there’s sometimes a backlash from doing that, but just … I put it out there”. Theoretically speaking, one wonders why his early and forthcoming disclosures did not result in more immediate linkage to care.

Those who delayed entry into care mirrored their engaged counterparts in that most disclosed to only a few, thus they were selective. In line with the theory, none of the delayed participants reported disclosing within the first year after diagnosis. Those that discussed disclosure timing tended to delay disclosure in line with their delayed entry into care.

### Inconsistent

Where the theory appeared supported among participants who were inconsistent in their care regarded disclosure to other PWH. One participant stated, “I have two childhood friends that they’re in the same boat [HIV positive]. We see each other whenever somebody has an appointment, then we talk, eat lunch … ” Another inconsistent participant stated, “My closest friends … are also infected … there’s a sort of brotherhood … ‘Oh, I know about such and such place where you can go get this or that, you can get help’”. On close inspection, however, these stories do not support the assertion that disclosure and social support mediates care engagement given the participants have been in and out of care since diagnosis, or, their stories suggest that social support’s influence comes and goes over time and experience.

Another inconsistent participant informed the limits of social support.

I hadn’t told her [a friend] and my partner took the liberty of telling her about my, our situation because he is also positive, and she changed her attitude. I go to her home and she’s always kind of taking care of things in the bathroom … there are things there that people, sometimes, I cannot criticize them, but clearly they are not well-informed because you do not get infected for using the bathroom … she’s disgusted by me, and probably, you know, if she were informed things would be reversed.

This narrative supports the complex and dynamic nature of social support. Not everyone will respond supportively, regardless of expectation. Thus, the roles involved in the support network do not always offer the assistance needed to sustain medical care engagement.

Regarding the theoretical assumptions for inconsistent PWH – that they have told very few people (thus receive less support) – one person cycling in and out of care stated, “I actually don’t care who I talk to about my HIV status”. Another participant stated, “My family knows, my mom, my brothers, my father, they all know … from the beginning”. These narratives refute the disclosure aspect of the theory.

Consistent with the engaged and delayed group, one-third of the inconsistent participants reported disclosing to four or more person roles. This affirms the suggestion that the number of people one discloses may not influence outcomes. The experiences of the five inconsistent participants who reported disclosing right after diagnosis and the five who disclosed for assistance in managing care do not support the notion that disclosure leads to social support or medical care engagement. These men did disclose, and several for support purposes, yet their inconsistency in care suggest that the support needed to remain in care did not manifest.

### Detached

Four participants presented as detached from HIV care. According to the theory’s logic, we would expect them to have refrained from disclosing. This was not the case.

It’s been easy in that area [disclosure] cause like my mother, you know, I’m very open with her … she’s understanding, and she’ll accept me no matter what … like my sisters and cousins and other relatives, uh, they’re aware of it too, that I’m positive.

Additionally, two of the four men had disclosed to their children, and all four disclosed to other family members. Two of the men had romantic partners, and both partners were aware of the participant’s status. At the same time, while these men disclosed, they leaned toward disclosing to fewer person roles compared to their counterparts. Ultimately, these four men had people in their lives that knew their status, thus presenting opportunities for social support to counter their detachment from care. In addition, while none of the four men reported disclosing shortly after diagnosis, one man reported seeking assistance for managing his care; however, he also reported his detachment from care.

## Discussion

The findings suggest that disclosure and social support did not uniformly facilitate or impede linking to or sustaining engagement in care. Again, we observed no pattern that affirmed or refuted the theory. Our findings lend support to the argument that disclosure and social support’s role in improving care is complex (Thoits, 1982, 1995). We posit that disclosure is undertaken in a specific context and for specific reasons. As such, disclosure depends on circumstances mediated by other factors, such as supportive friends and family, resilience and coping skills, mental illness and co-morbid health issues, stressors, structural disparities, and stigma. For some participants, disclosure occurred to fulfill a specific need or was relationship dependent, such as ensuring their boss understood why they call in sick. Yet for others, disclosure did not present as a reasonable option in their relationships, or at times in their lives.

Our findings support arguments that emphasize the likely importance of intention and the complexities of the context PWH navigate when they disclose their status or receive support. These findings underscore research that seeks to revise the dominant narrative of a simplified disclosure-to-social-support paradigm. For instance, Dima et al. (2014) posit that disclosure is influenced by the PWH’s intent of disclosing, the type of confidant, and the role of variables such as stigma. Dima and colleagues envision disclosure as a multidimensional and multi-actor process. For example, stigma related to HIV may affect disclosure differently depending on the age, socio-demographic background, and characteristics (mental health or substance use) of the PWH and the confident, as well as the disclosure environment (structural and institutional stigma). These factors make each act of disclosure unique with different consequences. Related, the Disclosure Process Model is a decision-making framework that argues for a more complex read of disclosure and social support when attempting to harness their potential (Chaudoir & Fisher, 2010; Chaudoir et al., 2011). The Disclosure Process Model includes specific goals (to disclose or not based on experience and environment), type of need (emotional, informational, or instrumental), the disclosure event (accounting for the confidant’s reaction as well as the environment), mediating processes (changes in social information, social support, and alleviation of individual inhibition), and long-term individual (sustained engagement), dyadic (decreases in sexual risk behavior), and social-contextual (increases in HIV awareness) factors. If we consider these frameworks, the men in our sample may be engaged in multi-dimensional decision-making at any stage of a disclosure process independent of time since diagnosis, and this may explain the variability in our findings.

The complexity of disclosure and social support, as evidenced by inconsistency in the literature and our findings, suggests that public health and medicine needs to identify a new paradigm that addresses the complexity when targeting HIV outcomes (Chaudoir & Fisher, 2010; Chaudoir et al., 2011; Dima et al., 2014). We propose five actions when promoting disclosure for social support reasons:

1. Identify intent – does the PWH have a specific need for emotional, instrumental, or informational support, emergency help, or to protect self or others.
2. Identify a confidant – family, friend, or peer who is likely to provide the intended support.
3. Identify a strategy – include expectations around privacy, decide how much to disclose, make HIV care needs specific (e.g., “I need advice on whether to go to the ER”).
4. Identify skills to counter stigma and misinformation, including how to recognize it, create teachable moments, and how to remove oneself from volatile situations.
5. Reassess support needs on a routine basis and discuss with the identified confidant.

This study has limitations. This was a purposefully selected sample of urban-residing black and Latino MSM living with HIV; as a result, their experiences may not be the same as other cohorts of PWH. As such, the findings are not representative. Further, given the study was on barriers and facilitators to HIV care and treatment, broadly conceived, we are not able to reflect on critical areas of disclosure and social support, such as the impact of evolving relationships and whether such evolutions altered the participants’ experiences with support, as well as care engagement. Finally, we conceived these analyses post hoc, thus important features, including key concept definitions, were not built into the interview guide. Participants may have understood *disclosure* and *social support* differently.

In conclusion, the participants offered limited support for the theorized association of disclosure and social support to care engagement. The data asks us to better understand the PWH-level details when attempting to utilize disclosure and social support toward improved outcomes – to whom, under what conditions, toward what end, and as a moment-in-time and longitudinal process. Without such an understanding, we may not be providing PWH, especially black and Latino MSM, the needed tools to manage disclosure and social support. Adapting interventions to inspect the dynamic context in which disclosure and social support occur may improve health-related outcomes.

## Figures and Tables

**Table 1.** Participant characteristics (*N* = 84).^a^

Characteristic	N	Percent
*City of residence*		
Atlanta	24	28.6
Baltimore/Washington, DC	17	20.2
Chicago	23	27.4
Los Angeles	20	23.8
*Age in years* ^b^		
20–29	12	15.0
30–39	16	20.0
40–49	27	33.8
50–59	25	31.3
	*Standard deviation*	*Range*
	10.3	20–59
*Race/ethnicity*		
Black/African American	50	59.5
Hispanic/Latino	31	36.9
Black/Hispanic-Latino	3	3.6
*Self-identified sexuality* ^c^		
Gay/homosexual	58	69.0
Bisexual	17	20.2
“Something else” or other response	9	10.7
*Level of education*		
<High school diploma	16	19.0
High school diploma/GED	19	22.6
Some college	32	38.1
>College graduate	17	20.2
*Health coverage*		
No coverage	20	23.8
Partial coverage, e.g., ADAP/Ryan White	15	17.9
Full coverage, e.g., Medicaid, medicare, private insurance	49	58.3
*HIV diagnosis pre- or post-ART availability*		
1980–1996	27	32.1
1997–2014	56	66.7
Unknown	1	1.2
*Care engagement status*		
Engaged	37	44.0
Delayed	15	17.9
Inconsistent	28	33.3
Detached	4	4.8

a Percent totals may not equal 100% due to rounding.

b Missing data on four participants (*N* = 80).

c Screened eligibility criteria included having had sex with another man in the prior six months.

## References

[R1] Centers for Disease Control and Prevention. (2017). Monitoring selected national HIV prevention and care objectives by using HIV surveillance data – United States and 6 dependent areas, 2015. HIV Surveillance Supplemental Report, 22(2).

[R2] Chaudoir SR, & Fisher JD (2010). The disclosure processes model: Understanding disclosure decision making and post-disclosure outcomes among people living with a concealable stigmatized identity. Psychological Bulletin, 136(2), 236. 20192562 10.1037/a0018193 PMC2922991

[R3] Chaudoir SR, Fisher JD, & Simoni JM (2011). Understanding HIV disclosure: A review and application of the disclosure processes model. Social Science & Medicine, 72(10), 1618–1629. 21514708 10.1016/j.socscimed.2011.03.028 PMC4059828

[R4] Christopoulos Katerina A., Das Moupali, & Colfax Grant N. (2011). Linkage and Retention in HIV Care among Men Who Have Sex with Men in the United States. Clinical Infectious Diseases, 52(suppl_2), S214–S222. doi:10.1093/cid/ciq045 21342910 PMC3106256

[R5] Cook CL, Canidate S, Ennis N, & Cook RL (2018). Types and delivery of emotional support to promote linkage and engagement in HIV care. Patient Preference and Adherence, 12, 45–52. doi:10.2147/ppa.s145698 29343948 PMC5749556

[R6] Crawford TN, & Thornton A (2017). Retention in continuous care and sustained viral suppression: Examining the association among individuals living with HIV. Journal of the International Association of Providers of AIDS Care, 16 (1), 42–47. doi:10.1177/2325957416678929 27852944

[R7] Dasgupta S (2016). Disparities in consistent retention in HIV care – 11 states and the District of Columbia, 2011–2013. Morbidity and Mortality Weekly Report, 65, 77–82. 26844978 10.15585/mmwr.mm6504a2

[R8] Daskalopoulou M, Lampe FC, Sherr L, Phillips AN, Johnson MA, Gilson R, … Rodger AJ (2017). Non-disclosure of HIV status and associations with psychological factors, ART non-adherence, and viral load non-suppression among people living with HIV in the UK. AIDS and Behavior, 21(1), 184–195. doi:10.1007/s10461-016-1541-4 27586375 PMC5216090

[R9] Dima AL, Stutterheim SE, Lyimo R, & de Bruin M (2014). Advancing methodology in the study of HIV status disclosure: The importance of considering disclosure target and intent. Social Science & Medicine, 108, 166–174. 24641881 10.1016/j.socscimed.2014.02.045

[R10] Elopre L, Hook EW, Westfall AO, Zinski A, Mugavero MJ, Turan J, & Van Wagoner N (2015). The role of early HIV status disclosure in retention in HIV care. AIDS Patient Care and STDs, 29(12), 646–650. 26588053 10.1089/apc.2015.0205 PMC4684646

[R11] Geter A, Sutton MY, & Hubbard McCree D (2018). Social and structural determinants of HIV treatment and care among black women living with HIV infection: A systematic review: 2005–2016. AIDS Care, 30(4), 409–416. doi:10.1080/09540121.2018.1426827 29376409 PMC6459180

[R12] Hall HI, Tang T, Johnson AS, Espinoza L, Harris N, & McCray E (2016). Timing of linkage to care after HIV diagnosis and time to viral suppression. Journal of Acquired Immune Deficiency Syndromes, 72(2), e57–e60. 26977745 10.1097/QAI.0000000000000989 PMC8666969

[R13] Hamilton MM, Razzano LA, & Martin NB (2007). The relationship between type and quality of social support and HIV medication adherence. Journal of HIV/AIDS & Social Services, 6(1–2), 39–63.

[R14] Daniel Kelly, J., Christine Hartman, Graham James, Kallen Michael A., & Giordano Thomas P. (2014). Social Support as a Predictor of Early Diagnosis, Linkage, Retention, and Adherence to HIV Care: Results From The Steps Study. Journal of the Association of Nurses in AIDS Care, 25(5), 405–413. doi:10.1016/j.jana.2013.12.002 PMC4125558 24508174

[R15] Laffoon BT, Hall HI, Babu AS, Benbow N, Hsu LC, Hu YW, & Workgroup UAHS (2015). HIV infection and linkage to HIV-related medical care in large urban areas in the United States, 2009. Journal of Acquired Immune Deficiency Syndromes, 69(4), 487–492. 25844695 10.1097/QAI.0000000000000619 PMC11328915

[R16] Liau A, Crepaz N, Lyles CM, Higa DH, Mullins MM, DeLuca J, … Marks G (2013). Interventions to promote linkage to and utilization of HIV medical care among HIV-diagnosed persons: A qualitative systematic review, 1996–2011. AIDS and Behavior, 17(6), 1941–1962. doi:10.1007/s10461-013-0435-y 23456593 PMC5906800

[R17] Mellins CA, Kang E, Leu C-S, Havens JF, & Chesney MA (2003). Longitudinal study of mental health and psychosocial predictors of medical treatment adherence in mothers living with HIV disease. AIDS Patient Care and STDs, 17(8), 407–416. 13678542 10.1089/108729103322277420

[R18] Nakigozi G, Makumbi FE, Bwanika JB, Atuyambe L, Reynolds SJ, Kigozi G, … Serwadda D (2015). Impact of patient-selected care buddies on adherence to HIV care, disease progression and conduct of daily life among pre-antiretroviral HIV-infected patients in Rakai, Uganda: A randomized controlled trial. Journal of Acquired Immune Deficiency Syndromes, 70(1), 75. 26039929 10.1097/QAI.0000000000000710 PMC4556592

[R19] Obermeyer CM, Baijal P, & Pegurri E (2011). Facilitating HIV disclosure across diverse settings: A review. American Journal of Public Health, 101(6), 1011–1023. 21493947 10.2105/AJPH.2010.300102 PMC3093267

[R20] Overstreet NM, Earnshaw VA, Kalichman SC, & Quinn DM (2013). Internalized stigma and HIV status disclosure among HIV-positive black men who have sex with men. AIDS Care, 25(4), 466–471. 23006008 10.1080/09540121.2012.720362 PMC4270468

[R21] Robertson M, Laraque F, Mavronicolas H, Braunstein S, & Torian L (2015). Linkage and retention in care and the time to HIV viral suppression and viral rebound – New York City. AIDS Care, 27(2), 260–267. doi:10.1080/09540121.2014.959463 25244545

[R22] Singh S, Mitsch A, & Wu B (2017). HIV care outcomes among men who have sex with men with diagnosed HIV infection-United States, 2015. Morbidity and Mortality Weekly Report, 66(37), 969–974. 28934185 10.15585/mmwr.mm6637a2 PMC5657781

[R23] Smith R, Rossetto K, & Peterson BL (2008). A meta-analysis of disclosure of one’s HIV-positive status, stigma and social support. AIDS Care, 20(10), 1266–1275. 18608080 10.1080/09540120801926977

[R24] Strachan ED, Bennett WRM, Russo J, & Roy-Byrne PP (2007). Disclosure of HIV status and sexual orientation independently predicts increased absolute CD4 cell counts over time for psychiatric patients. Psychosomatic Medicine, 69(1), 74–80. 17167125 10.1097/01.psy.0000249900.34885.46

[R25] Thoits PA (1982). Life stress, social support, and psychological vulnerability: Epidemiological considerations. Journal of Community Psychology, 10(4), 341–362. 10298894 10.1002/1520-6629(198210)10:4<341::aid-jcop2290100406>3.0.co;2-j

[R26] Thoits PA (1995). Stress, coping, and social support processes: Where are we? What next? Journal of Health and Social Behavior, Extra issue(Forty years of medical sociology: the state of the art and directions for the future), 53–79. 7560850

[R27] Thompson MA, Mugavero MJ, Amico KR, Cargill VA, Chang LW, Gross R, … Bartlett JG (2012). Guidelines for improving entry into and retention in care and antiretroviral adherence for persons with HIV: Evidence-based recommendations from an International Association of Physicians in AIDS Care panel. Annals of Internal Medicine, 156(11), 817–833. 22393036 10.7326/0003-4819-156-11-201206050-00419 PMC4044043

[R28] Valle M, & Levy J (2009). Weighing the consequences self-disclosure of HIV-positive status among African American injection drug users. Health Education & Behavior, 36(1), 155–166. 18697884 10.1177/1090198108316595

[R29] Waddell EN, & Messeri PA (2006). Social support, disclosure, and use of antiretroviral therapy. AIDS and Behavior, 10(3), 263–272. 16496089 10.1007/s10461-005-9042-x

[R30] Wohl Amy Rock, Galvan Frank H., Myers Hector F., Garland Wendy, George Sheba, Witt Mallory, & Cadden Joseph. (2011). Do Social Support, Stress, Disclosure and Stigma Influence Retention in HIV Care for Latino and African American Men Who Have Sex with Men and Women? AIDS and Behavior, 15(6), 1098–1110. doi:10.1007/s10461-010-9833-6 20963630

[R31] World Health Organization. (2013). HIV and adolescents: Guidance for HIV testing and counselling and care for adolescents living with HIV: Recommendations for a public health approach and considerations for policy-makers and managers. ANNEX 4, Systematic Review – ALHIV: Disclosure, Adherence and Retention in Care. 25032477

